# Optimal Replacement of Fish Meal Protein by Stick Water in Diet of Sex-Reversed Nile Tilapia (*Oreochromis niloticus*)

**DOI:** 10.3390/ani9080521

**Published:** 2019-08-02

**Authors:** Uraiwan Wattanakul, Wattana Wattanakul, Karun Thongprajukaew

**Affiliations:** 1 Department of Food Industry and Fisheries Products, Faculty of Sciences and Fisheries Technology, Rajamangala University of Technology Srivijaya, Trang 92150, Thailand; 2 Department of Fisheries Technology, Faculty of Sciences and Fisheries Technology, Rajamangala University of Technology Srivijaya, Trang 92150, Thailand; 3 Department of Applied Science, Faculty of Science, Prince of Songkla University, Songkhla 90112, Thailand

**Keywords:** carcass, digestive enzyme, feed utilization, fish condensate, growth, hematological parameter, histopathology, industrial waste, protein replacement

## Abstract

**Simple Summary:**

Only limited information is available on the use of stick water in aquafeed, even though this could confer benefits by providing cost-effective nutrition to aquaculture as well as reducing waste effluents. Therefore, the optimal replacement of protein from fish meal by stick water in the diet of Nile tilapia was investigated in the current study. An 8 month trial was conducted in floating baskets, mimicking the market stage Nile tilapia production in Thailand. Based on the overall results, 20% protein replacement of fish meal by stick water was near optimal, providing superior traits of overall observed parameters relative to the baseline fish meal-based diet. Findings from the current study suggest that stick water is a suitable alternative ingredient for sex-reversed Nile tilapia. The pursuit of higher replacement levels while maintaining the carcass quality of reared fish could be a topic for further study.

**Abstract:**

The effects of replacing fish meal (FM) protein with stick water (SW) were investigated during the market stage of sex-reversed Nile tilapia, *Oreochromis niloticus* (18.49 ± 0.31 g initial body weight). The FM protein was replaced with SW for 10% (10SW), 20% (20SW), 30% (30SW) and 50% (50SW) of the FM. The completely randomized design was conducted in outdoor 15 floating baskets (1.5 × 1.5 × 2 m), comprising three replications with 50 fish each, over an 8 month trial. At the end of the experiment, no differences in survival, growth performance or feed utilization were observed across the dietary treatments (*p* > 0.05). A significant change in lipase-specific activity was caused by the replacement, without changes to trypsin, chymotrypsin or amylase activities. The fish in all dietary groups exhibited normal liver histopathology, but the fish fed a diet containing SW showed higher numbers of cells accumulating lipids as compared to fish fed the baseline 0SW dietary treatment. Hematological parameters were similar across the five dietary groups. Only fish fed the 20SW diet had superior carcass quality compared to the baseline 0SW group, in terms of crude protein and lipids, but lower or higher replacement levels had negative effects on carcass quality. Findings from the current study support the replacement of FM protein with SW at a level of 20% in the diet of sex-reversed Nile tilapia reared to the market stage. Higher replacement levels might be possible with the supplementation of fatty acids.

## 1. Introduction

Nile tilapia (*Oreochromis niloticus*) is among the most important fish species in economies around the world. The production of tilapia (all species) increased from 2.5 million tonnes in 2010 to 4.2 million tonnes in 2016 [1]. Despite the high demand for these fish, their rearing is negatively impacted not only by the escape of this highly invasive fish into natural waters but also by fluctuations in the cost of feedstuffs used in their diet preparation. Fish meal (FM) is the most important protein source in aquafeed production. Increasing demand for supporting global aquaculture has caused increases in FM prices in the recent years. The replacement of protein from the costly FM with other alternative sources has been optimized in order to reduce the cost of feed production. However, such replacements at suboptimal levels may cause unbalanced amino acids, resulting in low feed utilization efficiency and poor growth [2,3,4,5].

Stick water (SW) or steam condensate is a byproduct from the marine canned seafood industry, sometimes also obtained from the manufacturing of FM and fish oil. It is prepared by cooking raw materials with steam, pressing them to squeeze out moisture and then evaporating the liquid extracts to obtain viscous, fast-decomposing and evil-smelling liquor. Generally, SW contains large amounts of protein, lipid and ash [6,7]. In applications to aquaculture, SW has been used as an attractant in aquafeed [8]. Only limited information is available on the use of SW in aquafeed, though it use could confer benefits by providing cost-effective nutrition to aquaculture as well as reducing waste effluents [7,9]. However, the replacement of protein from FM with SW may have negative effects on feed utilization due to the difference in amino acid profiles [7] or possibly due to the unsuitable composition of fatty acids.

In order to assess the performance of aquafeed with replaced ingredients, the nutritional responses through digestive enzyme activities have been reported in several prior studies [5,6,7,10,11,12,13]. In the current study, the optimal replacement of protein from FM with SW in the diet of Nile tilapia was investigated. Mono-sex males were used since they provide desirable characteristics for culture such as fast growth and high fillet quality, relative to mixed-sex or all-female fish [14,15]. An 8 month trial was conducted in floating baskets, mimicking the market stage Nile tilapia production in Thailand. Carcass composition is an important criterion in the evaluation of the quality of consumed fish. Long-term side effects were also investigated by hematological parameters and liver histopathology. Findings from the current study suggest an optimal protein replacement of FM with raw SW without any supplementation.

## 2. Materials and Methods

All feedstuffs were purchased from a private sector in the province of Phatthalung, Thailand. Raw SW was obtained from a canned seafood factory (Kuang Pei San Food Products Public CO., Ltd., Trang, Thailand). This byproduct is a condensate from the steam conditioning of mackerels (including *Decapterus macarellus, Rastrelliger brachysoma*, *R. faughni*, *R. kanagurta* and *Scomber australasicus*), which is then boiled until constant total soluble solids are obtained (57–60°Bx and ≤400 g kg^−1^ moisture content). The chemical compositions of the main feedstuffs, including FM, SW, soybean meal (SBM), rice bran, corn meal and broken rice, are shown in Table 1, while the fatty acid composition of the SW is shown in Table 2. The four experimental diets were designed to partly replace the FM protein with SW at levels of 10% (10SW), 20% (20SW), 30% (30SW) and 50% (50SW) (Table 3). The FM-based diet (0SW) was used as a control. All solid feedstuffs were ground, sieved, weighed and then mixed for 10 min. Soybean oil, fish oil and vitamin-mineral premixes were added, followed by 300 g kg^−1^ water, to obtain a dough-like consistency. The experimental diets (2–4 mm die diameter) were produced after pelleting through a meat mincer. The pellets were then dried at 60 °C for 12 h, cut and sieved. The pellets were packed in polyethylene bags, sealed and stored in a refrigerator (4 °C) until they were used in feeding.

The proximate chemical compositions of the main feedstuffs and all experimental diets were analyzed according to standard methods of AOAC [16]. Nitrogen-free extract (NFE, g kg^−1^) and gross energy (GE, kcal kg^−1^) were calculated from 1000−(CP + crude lipid + crude ash + crude fiber) and (CP × 5.6) + (crude lipid × 9.44) + (crude fiber × 4.1) + (NFE × 4.1), respectively. For the SW component, its fatty acid profile was also determined. The fatty acid methyl esters were prepared using BF_3_ in methanol, and the fatty acid profile was analyzed using a gas chromatograph (Agilent 6890; Agilent Technologies Inc., Santa Clara, CA, USA) equipped with a flame ionization detector. The split injection temperature was set at 270 °C. The detector temperature was maintained at 250 °C, the hydrogen flow at 40 mL min^−1^ and the air flow at 450 mL min^−1^. High-purity helium was used as the carrier gas at 2 mL min^−1^. Menhaden oil methyl esters (National Marine Fisheries Service, Seattle, WA, USA) were used as internal standards.

Seed production of Nile tilapia was conducted at Phatthalung Inland Fisheries Research and Development Center, Phatthalung, Thailand. The fish fry were fed the diet containing 40 mg kg^−1^ 17-α-methyltestosterone for three weeks to induce mono-sex males, ≥99% based on acetocarmine staining [17]. After that, sex-reversed fish were transported to the Department of Fisheries Technology, Faculty of Sciences and Fisheries Technology, Rajamangala University of Technology Srivijaya, Trang, Thailand, where they were acclimatized in a cement pond (1 × 4 × 1 m) for 10 days. They were fed to satiation with the control diet (0SW) at 08.00 and 16.00 h daily under natural light conditions (lights on 06.00 to 18.00 h). Subsequently, fish with similar weight (18.49 ± 0.31 g initial body weight) were distributed into 15 floating baskets (1.5 × 1.5 × 2 m) in a 1600 m^2^ pond. The baskets were covered by 1 inch black straight seine during the first three months, and these were changed to 1.2 inch size seine until the end of the experiment. Each treatment included a total of 150 fish for each replication. The experiment was conducted for 8 months. The fish were fed ad libitum, and the uneaten feed was collected 1 h after feeding, dried at 60 °C until constant weight and the determined weight was used to calculate the feed intake (FI), the feed conversion ratio (FCR) and the protein efficiency ratio (PER). The measured growth and the feed utilization were summarized monthly. At the end of the experiment, all fish were starved for 24 h prior to measuring body weight (BW) and length, followed by collecting samples of whole carcass, intestine and blood. All uses of animals in the current study conformed to the “Ethical Principles and Guidelines for the Use of Animals for Scientific Purposes”, National Research Council, Thailand (Application No. U1-06514-2560).

Growth and feed utilization parameters of reared fish were calculated as follows:

Survival (%) = 100 × [final fish number/initial fish number],

Condition factor (CF, g cm^−3^) = 100 × [live BW (g)/total body length (cm)^3^],

Stomasomatic index (SSI, %) = 100 × [wet weight of stomach (g)/wet BW (g)],

Intestosomatic index (ISI, %) = 100 × [wet weight of intestine (g)/wet BW (g)],

Specific growth rate (SGR, % BW day^−1^) = 100 × [(lnW_t_ − lnW_0_)/(t − t_0_)],

where W_t_ = mean weight (g) at day t and W_0_ = mean weight (g) at day t_0_.

FI (g day^−1^) = F/(W_0_ + W_1_/2)(N_0_ + N_1_/2)t,

where F = dry feed fed (g), W_0_ = average initial BW (g),

W_1_ = average final BW (g), N_0_ = initial fish number, N_1_ = final fish number and t = rearing period (day).

FCR (g feed g gain^−1^) = dry feed consumed (g)/wet weight gain (g),

PER (g gain g protein^−1^) = wet weight gain (g)/protein intake (g).

The quality of water was measured every other week. The temperature, pH, total alkalinity and total ammonia were determined according to standard methods of APHA, AWWA and WPCF [18]. Nitrite was determined according to the method of Strickland and Parsons [19]. Dissolved oxygen was determined using a Multiparameter Display System (YSI 650MDS, YSI Incorporated, Yellow Springs, OH, USA). The water quality parameters during the experiment were in the ranges of 28.91 ± 0.30 °C, pH 7.15 ± 0.08, 6.72 ± 0.19 mg L^−1^ dissolved oxygen, 98.95 ± 1.25 mg L^−1^ alkalinity, 0.41 ± 0.02 mg L^−1^ ammonia and 0.24 ± 0.02 mg L^−1^ nitrite.

The fish were anesthetized with tricainemethanesulfonate (MS-222). The blood samples were collected (*n* = 3 per replication) with a 2 mL syringe from the caudal vessel. Plasma protein was determined according to the Lowry method [20]. Red (RBC) and white (WBC) blood cell counts from diluted samples were determined [21]. Hemoglobin (Hb) and hematocrit (Hct) were assayed as described in the work of Larsen and Snieszko [22]. Blood indices in terms of mean cell volume (MCV), mean cell hemoglobin (MCH) and mean cell hemoglobin concentration (MCHC) were calculated as follows [23]:

MCV (mm^3^) = [Hct (%)/RBC (×10^6^ µL)] × 10,

MCH (pg cell^−1^) = [Hb (g dL^−1^)/RBC (×10^6^ µL)] × 10,

MCHC (g L^−1^) = Hb (g dL^−1^)/Hct (%).

The liver samples (*n* = 3 per replication) were gently rinsed with cold water, fixed in 10% phosphate-buffer formalin, dehydrated by standard procedures, embedded in paraffin, sliced to sections with a rotary microtome (Shandon AS325 Retraction; Life Sciences International, Cheshire, UK) and stained with hematoxylin and eosin (H&E). Histopathological examination was conducted under a light microscope at 400× magnification.

The intestine with food content (*n* = 3 per replication) was removed onto ice, dissected and then homogenized in 0.2 M Na_2_HPO_4_-NaH_2_PO_4_ buffer (pH 8, 1:3 w/v). Centrifugation of tissue homogenate was performed at 15,000× *g*, at 4 °C for 30 min to obtain the supernatant. This crude enzyme extract was kept in aliquots at −20 °C until use. The protein concentration of a crude enzyme extract was determined using the Lowry method [20]. Bovine serum albumin (BSA) was used to construct the standard curve. The activities of trypsin (EC 3.4.21.4) and chymotrypsin (EC 3.4.21.1) were assayed using *N*-benzoyl-*L*-Arg-*p*-nitroanilide (BAPNA) and *N*-succinyl-Ala-Ala-Pro-Phe-*p*-nitroanilide (SAPNA) as the substrates, respectively [24]. Amylase activity (EC 3.2.1.1) was assayed using starch soluble as the substrate [25]. Lipase activity (EC 3.1.1.3) was assayed using *p*-nitrophenyl palmitate as a substrate [26].The measured products of these four enzymes were referenced to the concentrations of *p*-nitroanilide (A_410_), *p*-nitroanilide (A_410_), maltose (A_540_) and *p*-nitrophenol (A_410_), respectively, to quantify their activities.

The whole bodies (*n* = 3 per replication), excluding the intestine, were minced and used to determine the proximate chemical compositions. The moisture, CP, crude lipid and ash were analyzed according to standard methods of AOAC [16].

Fifteen experimental units were set under a completely randomized design (five treatments × three replications). All the data are herein expressed as the mean ± standard error of the mean (SEM). All statistical evaluations were made using SPSS Version 14 (SPSS Inc., Chicago, IL, USA). Variables that are percentages were subjected to arcsine transformation. One-way ANOVA was used to check differences between the treatments and then Duncan’s multiple range test was used to confirm significant differences. The significance criterion was set at *p* < 0.05 in all statistical analyses.

## 3. Results

### 3.1. Fatty Acid Profile of SW and Chemical Composition of the Experimental Diets

The raw material, on a dry weight basis, contained 48.31 g kg^−1^ saturated fatty acids (SFA), 9.62 g kg^−1^ monounsaturated fatty acids (MUFA), 65.84 g kg^−1^ polyunsaturated fatty acids (PUFA), 40.60 g kg^−1^ long chain-PUFA (LC-PUFA), 39.28 g kg^−1^ *n*–3 and 25.18 g kg^−1^ *n*–6, giving the *n*–3:*n*–6 ratio 1.56 (Table 2). All the experimental diets were similar in proximate chemical compositions (Table 3). The experimental diets were mutually isonitrogenous, isolipidic and isoenergetic.

### 3.2. Survival, Growth and Feed Consumption

Survival (97% on average) of reared fish did not differ across all the dietary treatments (*p* > 0.05, Table 4). The performances in terms of final BW, total length, CF, SSI, ISI, SGR, FI, FCR and PER were also similar across all treatments.

### 3.3. Hematological Parameters

At the end of the experiment, the levels of plasma protein (8.09 ± 0.20 g% on average), RBC (2.08 ± 0.09 × 10^6^ cells µL^−1^ on average), WBC (2.31 ± 0.05 × 10^4^ cells µL^−1^ on average), Hb (6.77 ± 0.12 g dL^−1^ on average), Hct (27.07 ± 0.49% on average), MCV (131.09 ± 5.77 mm^3^ on average), MCH (32.78 ± 1.44 pg cell^−1^ on average) and MCHC (250.04 ± 0.04 g L^−1^ on average) did not differ across the five dietary treatments.

### 3.4. Liver Histoarchitecture

**Figure 1:**
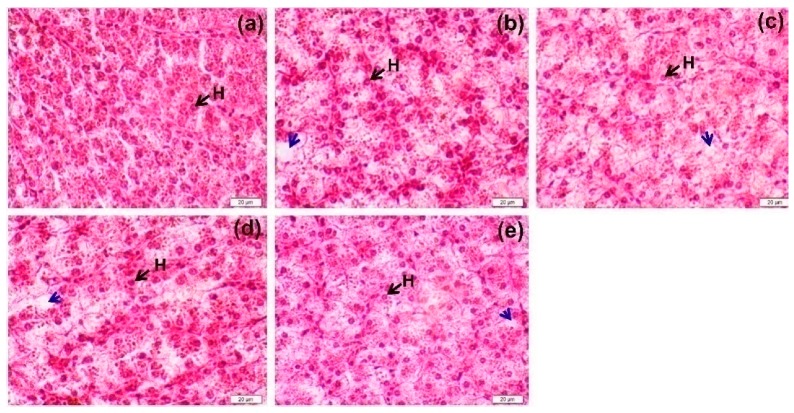
The liver histoarchitecture (longitudinal section) for sex-reversed Nile tilapia fed 0SW (**a**), 10SW (**b**), 20SW (**c**), 30SW (**d**) and 50SW (**e**) for 8 months. Tissues were stained with hematoxylin and eosin (H&E) and the images were photographed at 400× magnification. H = hepatocyte, blue arrows = cells accumulating lipids.

Fish from all treatments exhibited normal-shaped hepatocytes with regular gross morphology and clearly located cell nuclei (Figure 1). Compared to the control FM-based diet (Figure 1a), the number of cells accumulating lipids (blue arrows) appeared to increase in density with the increasing replacement level of FM protein by SW (Figure 1b–e). However, there were no signs of any kind of necrosis or inflammation in the liver histoarchitecture with any of the alternative dietary treatments.

### 3.5. Digestive Enzyme-Specific Activities

**Figure 2:**
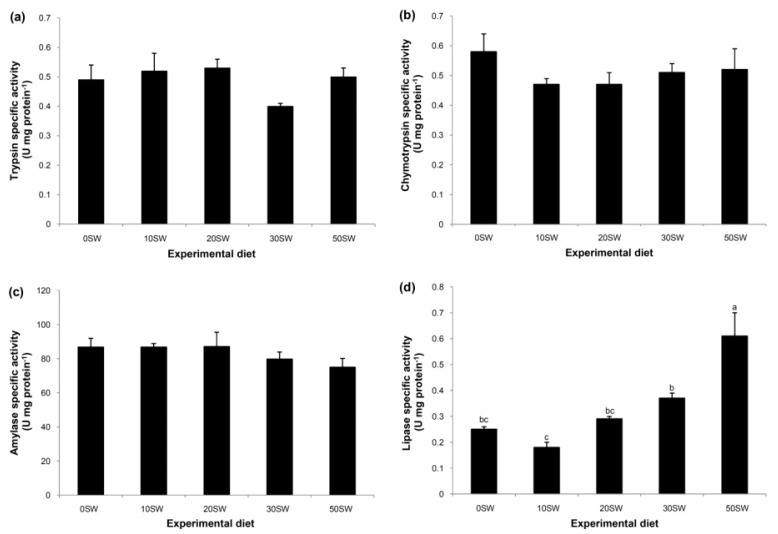
The specific activities of trypsin (**a**), chymotrypsin (**b**), amylase (**c**) and lipase (**d**) in sex-reversed Nile tilapia fed experimental diets containing various levels of FM protein replaced with SW. The data are the mean ± SEM (*n* = 3 per replication). Different superscripts are significantly different between groups (*p* < 0.05).

There were no effects of protein replacement in FM by SW on the specific activities of trypsin (Figure 2a), chymotrypsin (Figure 2b) or amylase (Figure 2c). The fish fed the 50SW diet had the highest lipase-specific activity, while the remaining treatments were mutually rather similar, with a significant difference only between the 10SW and the 30SW groups (Figure 2d).

### 3.6. Carcass Composition

The moisture and ash contents were unaffected by the alternative dietary treatments (Table 5). The CP was lowest in the fish fed the 30SW diet, while the other treatments gave mutually similar levels. The significantly decreased lipid content was found in fish fed the 10SW and 50SW diets relative to control FM-based diet.

## 4. Discussion

The water consumption by the precooking step in a canned seafood factory is about 0.5 m^3^ tonne^−1^ of fish raw material [27]. The generated wastewater is then evaporated to obtain a viscous, fast-decomposing and evil-smelling liquor, SW. Although SW contains large amounts of moisture, it is relatively high in CP, crude lipid and ash, while the crude fiber and NFE contents are very low [7,8]. The contents of essential amino acids (EAA) in SW are inferior as compared to FM, except for histidine [7], leading to different ratios of EAA to non-essential amino acids. Also, fatty acids are present in relatively lower amounts in SW compared to FM [28]. This deficiency in amino acids and fatty acids might not satisfy the nutritional requirements of fish, so the optimization of the inclusion level, or alternatively supplementation with synthetic nutrient sources, is necessary.

All the water quality parameters in the current study were within the standards for tilapia aquaculture [29]. The various replacement levels of protein in FM by SW had no effect on growth or feed consumption. The SGR (1.03% BW day^−1^ on average) may seem slightly low and the FCR (2.68 g feed g gain^−1^ on average) slightly high in the current study, relative to typical values reported. This is due to the current trial being long-term, with the larger fish having a higher feed consumption than the smaller fish. Generally, FM protein can be replaced by SW at levels of 40–50% in aquafeed, and in a 350–400 g kg^−1^ CP diet for giant freshwater prawn and striped snakehead [6,7]. Based on the survival, growth performance or feed utilization of reared fish in the current study, SW could replace FM protein up to the maximum value tested (50SW in a 300 g kg^−1^ CP diet).

Hematological examination could be used to trace the physiological or pathological changes of reared fish [30,31]. For Nile tilapia, there were no differences in the observed hematological parameters across the five dietary treatments, in contrast to changes in hematological parameters in fishes fed diets containing SBM-derived protein [4,5]. The observations following SBM inclusion might be caused by anti-nutritional compounds, such as phytohemagglutinins, affecting blood functionality [32]. The blood profiles observed in the current study are in agreement with the stable liver histoarchitectures observed, when fish fed diets containing SW were compared to the 0SW group. Similarly, a high replacement level of FM protein by SW can induce the accumulation of lipids in cells [6], while it does not affect body morphometry (CF) or visceral organ indices (SSI and ISI). This phenomenon is in agreement with use of animal protein blend instead of FM [3]. Since there were no changes in hematological parameters or liver histoarchitecture and survival was unaffected, this indicates a lack of toxic dietary effects from the protein replacement levels of the current study, during the long-term trial over 8months.

Trypsin and chymotrypsin are the main protein-digesting enzymes in the intestine of fish. Only trypsin can activate itself as well as other zymogens, such as chymotrypsinogen to chymotrypsin, contributing 40–50% of the overall protein digestion [33]. The activities of both these enzymes are important in the physiological responses to feed protein stressors [34]. Therefore, the stable activities of both these enzymes indicate unchanged protein digestion in the intestine, while the PER indicates steady protein utilization. Thus, tilapia appear to have the capacity to adjust their digestive proteases to a range of SW inclusion levels. Similar observations were reported when the protein in FM was replaced by SW in the range of 10–60% in the diet of freshwater prawns [7].

Carbohydrate utilization by aquatic animals has been widely studied by assessing the activity of α-amylase. This enzyme activity can be changed according to diet replacement in some aquaculture animals [11,12]. The finding of no difference in amylase activity between the treatments in the current study is in agreement with observations of hybrid tilapia fed various levels of SBM as a replacement of FM protein [10]. This suggests that tilapia may have the capacity to utilize carbohydrates in response to changes of inclusion level in the diet.

Lipase-specific activity increased linearly with the protein replacement level, suggesting that the low levels of digestible protein in the SW diet lead to protein sparing [35]. This assumption is based on three observations: the high CP and lipid contents in the SW diet; comparable CP but low lipid in fish carcasses that were fed a higher dose of SW; and lipase-specific activity.

No negative effect on carcass composition was observed only in tilapia fed 20SW, compared to the 0SW dietary baseline, while higher replacement levels exhibited significant effects on either CP or lipid levels. These findings match well with the significantly reduced accumulation of lipids in the carcass of striped snakehead that were fed diets with some protein replacement levels of FM by SW (20% and 40% CP) [6]. The optimal protein replacement level in the diet for tilapia (20SW in ca. 300 g kg^−1^ CP diet), based on the overall observations in the current study, is clearly below those reported in prior studies. The omnivorous feeding habit of Nile tilapia appears to affect its ability to utilize the nutrients in SW, with results different from the carnivorous striped snakehead. The findings from lipase-specific activity and the composition of carcass lipids in the current study indicate unbalanced lipid composition profiles, especially in terms of the fatty acids, due to the protein replacement of FM by SW. The supplementation of some select fatty acids with increasing protein replacement levels should be further investigated, even a few months prior to the catch, in order to improve the carcass compositions. In addition, from an economic viewpoint, the current prices of SW are very inexpensive in comparison to FM (0.45 vs. 0.84 USD kg^−1^); therefore, the 20SW diet (1.58 USD kg fish^−1^) compares well against the 0SW baseline diet (1.68 USD kg fish^−1^). This aspect supports the use of the low cost agro-industrial byproduct SW in the diet for sex-reversed Nile tilapia.

## 5. Conclusions

The replacement of FM protein by SW from 10 to 50% gave similar results in terms of the survival, growth and feed utilization of Nile tilapia, and had no negative effect on hematological or histopathological examinations over an 8 month dietary treatment at the market stage. Specific activities of trypsin, chymotrypsin and amylase indicated no change in the catabolism of protein or carbohydrate within this replacement range. However, changes in the specific activity of lipase and the carcass lipid and carcass protein contents significantly depended the replacement level. Based on the overall results, the 20SW diet was near optimal, maintaining carcass quality relative to the 0SW baseline diet. Findings from the current study suggest that SW is a suitable alternative ingredient for sex-reversed Nile tilapia. The pursuit of higher replacement levels while maintaining the carcass quality of reared fish could be a topic for further study. Additionally, the development of containment measures or escapement control to prevent this invasive fish from entering natural waters should be of interest.

## Figures and Tables

**Table 1:** Proximate compositions (g kg^−1^ on fresh weight) of the main feedstuffs in the experimental diets. The analyses were performed in triplicate.

Composition	FM	SW	SBM	Rice Bran	Corn Meal	Broken Rice
Moisture	53	402	90	76	90	126
CP	571	399	460	135	74	68
Crude lipid	80	128	13	146	47	3
Crude fiber	1	nd	74	67	22	5
Ash	174	71	74	74	26	4
NFE	121	nd	289	502	741	794
GE (kcal kg^-1^)	4453	3443	4187	4467	3986	3685

FM, fish meal; SW, stick water; SBM, soybean meal; CP, crude protein; nd, not detected; NFE, nitrogen-free extract; GE, gross energy.

**Table 2:** Fatty acid contents in SW (g kg^−1^ dry weight). The values are averaged from triplicate determinations.

Fatty Acid	Content (g kg^−^^1^)
C14:0	3.89
C15:0	1.61
C16:0	28.24
C17:0	2.29
C18:0	8.34
C20:0	0.90
C21:0	0.33
C22:0	0.47
C24:0	0.23
C16:1*n*–7	5.80
C18:1*n*–9	0.48
C18:2*n*–6	21.24
C18:3*n*–6	0.27
C18:3*n*–4	1.37
C18:4*n*–3	1.97
C20:1*n*–9	2.39
C20:2*n*–6	0.38
C20:3*n*–3	0.25
C20:3*n*–6	0.18
C20:4*n*–3	0.62
C20:4*n*–6	2.73
C20:5*n*–3	10.05
C22:1*n*–9	0.94
C22:5*n*–6	0.38
C22:5*n*–3	2.41
C22:6*n*–3	23.98
Ʃ SFA	48.31
Ʃ MUFA	9.62
Ʃ PUFA	65.84
Ʃ LC-PUFA	40.60
Ʃ *n*–3	39.28
Ʃ *n*–6	25.18
Ʃ *n*–3:*n*–6	1.56

SFA, saturated fatty acids; MUFA, monounsaturated fatty acids; PUFA, polyunsaturated fatty acids; LC-PUFA, long chain-PUFA.

**Table 3:** Ingredients and proximate compositions (g kg^−1^ of fresh weight) of the experimental diets used for rearing sex-reversed Nile tilapia.

Item	0SW	10SW	20SW	30SW	50SW
**Ingredient**					
FM	315	284	252	221	158
SW	0	40	80	120	200
SBM	190	191	192	195	199
Rice bran	118	116	113	109	102
Corn meal	118	116	113	109	102
Broken rice	118	116	113	109	102
Alpha starch	30	30	30	30	30
Soybean oil	30	30	30	30	30
Fish oil	46	46	46	46	46
Vitamin premix ^1^	15	15	15	15	15
Mineral premix ^2^	20	16	16	16	16
**Chemical composition**					
Moisture	63	62	63	66	68
CP	298	304	310	306	315
Crude lipid	134	158	174	156	163
Ash	114	116	105	104	106
Crude fiber	45	43	40	38	36
NFE	346	317	308	330	312
GE (kcal kg^−^^1^)	4537	4670	4805	4695	4730

FM, fish meal; SW, stick water; SBM, soybean meal; CP, crude protein; NFE, nitrogen-free extract; GE, gross energy. ^1^ Vitamin premix; 1 kg contained 12,000 U vitamin A, 2000 U vitamin D, 3000 mg vitamin E, 20 mg vitamin K, 100 mg thiamine HCl, 150 mg riboflavin, 500 mg niacin, 500 mg *D*-pantothenic acid, 25 mg pyridoxine HCl, 1 mg biotin, 5 mg folic acid, 125 mg vitamin B_12_, 1000 mg ascorbic acid, 1500 mg choline and 1 mg inositol. ^2^ Mineral premix; 1 kg contained 3 g NaCl, 1 g KCl, 1.4 g MgSO_4_, 0.2 g FeC_6_H_5_O_7_, 0.25 g MnSO_4_, 0.01 g KI, 0.13 g ZnCO_3_, 0.01 g CuSO_4_ and 6 g CaHPO_4_.

**Table 4:** Survival, growth performance and feed utilization of sex-reversed Nile tilapia fed experimental diets containing various levels of FM protein replaced with SW.

Parameter	0SW	10SW	20SW	30SW	50SW	*p*-Value
Survival (%)	98.00 ± 0.00	96.00 ± 2.00	98.00 ± 0.00	98.00 ± 0.00	96.00 ± 1.15	0.398
Final body weight (g)	231.26 ± 27.94	207.50 ± 8.28	235.06 ± 6.13	225.92 ± 7.92	223.64 ± 19.56	0.790
Final total length (cm)	19.52 ± 0.47	20.18 ± 0.92	21.09 ± 0.16	20.15 ± 0.58	19.31 ± 0.19	0.241
CF (g cm^−^^3^)	2.21 ± 0.06	2.07 ± 0.14	2.13 ± 0.10	2.08 ± 0.08	2.29 ± 0.20	0.623
SSI (%)	1.85 ± 0.43	1.87 ± 0.04	1.51 ± 0.03	1.65 ± 0.20	1.35 ± 0.15	0.758
ISI (%)	5.03 ± 0.13	5.24 ± 0.54	5.32 ± 0.29	5.44 ± 0.47	5.28 ± 0.78	0.983
SGR (% BW day^−1^)	1.04 ± 0.04	1.01 ± 0.01	1.05 ± 0.01	1.03 ± 0.01	1.03 ± 0.06	0.951
FI (g day^−^^1^)	0.021 ± 0.002	0.024 ± 0.001	0.021 ± 0.001	0.022 ± 0.001	0.023 ± 0.001	0.365
FCR (g feed g gain^−^^1^)	2.58 ± 0.21	2.93 ± 0.17	2.52 ± 0.04	2.65 ± 0.06	2.73 ± 0.16	0.370
PER (g gain g protein^−^^1^)	2.13 ± 0.37	1.82 ± 0.13	2.20 ± 0.06	2.02 ± 0.06	1.70 ± 0.08	0.316

SW, stick water; CF, condition factor; SSI, stomasomatic index; ISI, intestosomatic index; SGR, specific growth rate; BW, body weight; FI, feed intake; FCR, feed conversion ratio; PER, protein efficiency ratio. Different superscripts within the same row are significantly different (*p* < 0.05).

**Table 5:** Carcass proximate composition (g kg^−1^ wet weight) of the sex-reversed Nile tilapia fed experimental diets containing various replacement levels of FM protein by SW.

Carcass Composition	0SW	10SW	20SW	30SW	50SW	*p*-Value
Moisture	720 ± 32	723 ± 17	669 ± 22	694 ± 35	721 ± 32	0.612
CP	204 ± 6 ^a^	205 ± 6 ^a^	205 ± 2 ^a^	178 ± 6 ^b^	203 ± 4 ^a^	0.015
Crude lipid	59 ± 1 ^ab^	45 ± 1 ^c^	62 ± 1 ^a^	56 ± 1 ^b^	46 ± 2 ^c^	<0.001
Ash	33 ± 5	36 ± 2	31 ± 2	30 ± 5	30 ± 1	0.981

SW, stick water; CP, crude protein. Different superscripts within the same row are significantly different (*p* < 0.05).
